# Cumulative social adversity as a correlate of self harm: Validity of the Reward Probability Index

**DOI:** 10.1371/journal.pone.0326682

**Published:** 2026-03-13

**Authors:** Bella Magner-Parsons, Lee Hogarth

**Affiliations:** Department of Psychology, Washington Singer Laboratories, University of Exeter, United Kingdom; Chengdu Fifth People's Hospital, CHINA

## Abstract

Adversity is a risk factor for Non-Suicidal Self-Injury (NSSI). However, studies differ in their conceptualisation and indices of adversity, creating heterogeneity. The current study sought to validate the environmental adversity (ES) subscale of the Reward Probability Index (RPI) as a correlate of NSSI (and related risk factors: depression, anxiety, and impulsivity) to demonstrate the utility of this short ES questionnaire in self-harm research as a novel measure of cumulative social environmental adversity. A single, cross-sectional, online survey was completed by 149 participants, 50.3% of whom reported past year NSSI engagement. In adjusted models, environmental adversity (OR=3.8), depression (OR=1.1), low subjective socioeconomic status (SES) (OR=1.4) and indirect NSSI (OR=3.7) were associated with an increased odds of past year NSSI engagement. Pearson correlations within the NSSI subsample revealed environmental adversity, depression, and anxiety were associated with each other and NSSI, while impulsivity was not. Finally, a robust parallel mediation analysis indicated that the relationship between environmental adversity and NSSI was mediated by depression β = .165, 95%CI [.033,.336] (*R*^*2*^ = 76.87%), but not anxiety β = .017, 95%CI [−.143,.173] (*R*^*2*^ = 8.07%). These findings are consistent with empirical longitudinal and theoretical evidence proposing NSSI is associated with aversive environmental experience and depression. The finding that the environmental adversity subscale of the RPI is a valid correlate of NSSI, and is associated with other established risk factors for NSSI, validates the ES subscale for use in future longitudinal studies of NSSI as a short general assay of cumulative adversity.

## Introduction

Emphasising the diversity in adversity relevant to self-harm, a recent Lancet commission on self-harm stated:

…social, political, cultural, and ecological aspects of self-harm are often ignored, or are only superficially acknowledged, resulting in narrow interpretations of self-harm as a pathological sign of a psychiatric disorder. This individualising perspective might not sufficiently address social and structural drivers of pain and misery [1] (p. 1457).

Lifetime exposure to trauma, or adverse circumstances is estimated to affect ~70% of the population globally [2]. Broadly defined, trauma refers to any adverse experience which leads to distress which an individual cannot effectively cope with [3]. Trauma exposure and distress emerge as central components of theoretical conceptualisations and empirical investigations of self-injurious behaviours [4]. Self-injurious behaviours have gained increasing recognition as a significant clinical and global public health issue [5] associated with high costs to both individuals, and services [6]. Non-suicidal self-injury (NSSI) is a subset of injurious behaviours defined as deliberate, direct behaviour intended to harm the self through destruction of bodily tissue [7]. For example, skin cutting, picking, and self-hitting, in absence of intent to die [8]. These behaviours do not include socially sanctioned destruction of bodily tissue such as tattooing or piercing. Additionally, high-risk behaviours (e.g., reckless driving) and ‘indirect’ self-injury (e.g., provoking physical fights) are not classified as deliberate NSSI under this definition, as the consequences of these behaviours may be unintended, or vicarious (e.g., provoking an animal to harm oneself), rather than engaged in deliberately to cause oneself harm [9]. Prevalence estimates of NSSI vary considerably due to differences in sample characteristics, and methodological heterogeneity in assessing NSSI, with estimates ranging between 7.5–46.5% for general adolescent samples, 38.9% for university students, and 4–23% for general adult samples [4]. In light of evidence that NSSI prevalence has been increasing in young adults (18–25) over the last 20 years, further investigation into risk pathways of NSSI in young adult samples is needed [10].

Multiple risk models for NSSI exist in the literature, with varying theoretical perspectives aiming to explain why NSSI occurs. Psychiatric risk factors such as anxiety and depression have been identified in models such as the Experiential Avoidance Model (EAM) [11] where NSSI functions to relieve feelings of negative affect [12]. In more complex sequential models, distal factors such as the environment and genes are identified as origins of the risk pathway, leading to increased negative affect as part of a stress-response to the environment and subsequently, NSSI engagement (Integrated Model of NSSI) [13]. There is longitudinal evidence supporting increased NSSI risk from exposure to trauma including physical, sexual or emotional abuse, parental mental health problems, bullying and parental conflict [14], and negative affect, primarily depression [15] and anxiety [16]. Moreover, impulsivity has gained recognition as a potential risk factor for NSSI, and a mediator between the adverse experience – NSSI pathway [17,18]. However, comparatively few studies have assessed the unique contribution of individual risk factors proposed by different theoretical accounts of NSSI in a single study. It is important to test the unique relative contribution of proposed risk factors of NSSI within a single model, to increase understanding of the unique factors associated with NSSI engagement and hence to guide intervention and prevention strategies.

Individuals engaging in NSSI are a heterogenous group [19], though exposure to adverse environmental experiences (broadly defined) is so well-established in the literature it is considered a common risk factor [5]. Adverse Childhood Experiences (ACE’s), a set of exposures including physical, sexual, and emotional abuse, which occur in childhood, prior to the age of 18, are the focus of a substantial portion of the literature investigating predictors of NSSI [20,21]. While ACE’s are consistently associated with increased NSSI engagement [18] and wider poor health outcomes in adulthood [22], the reporting of ACE’s may be affected by recall bias in adult samples, given it relies on retrospective recall of events [23,24]. Moreover, there is recognition of significant heterogeneity in adverse experiences predicting subsequent NSSI engagement, with no single event explaining adequate variance in initiating the risk pathway [25]. Thus, investigation of recent adverse life events and ‘stressors’ has been assessed [26]. Exposure to unambiguously negative life stressors measured using the Life Events Checklist (LE-C) [27] (e.g., witnessing death, natural disasters, illness, physical and sexual assault) is prospectively positively associated with NSSI, suggesting more recent adverse experience may also contribute to NSSI engagement. Finally, adult exposure to physical and psychological abuse, and financial stress [28,29] are prospectively positively associated with NSSI. Given the plethora of adverse events associated with subsequent NSSI, broad-ranging measures of environmental adversities including assays of both event exposure and structural adversity may be beneficial when assessing factors associated with NSSI engagement.

As environmental adversity is multi-dimensional, researchers have been forced to choose between utilising a checklist of items that measures a subset of specific forms of adversity such as childhood experience of interpersonal violence, for example, the childhood experiences of violence questionnaire [30]; a checklist containing a larger number of specific forms to cover more instances, for example, the Adverse childhood experiences international questionnaire (ACE-IQ: [31]); or a smaller number of items that define environmental adversity more generally (non-specifically) but is quicker to complete for example, single-item subsets and brief scales such as the environmental suppressors (ES) subscale of the reward probability index (RPI) [32–34]. This latter approach, of aggregating scores across a relatively small number of items which define environmental adversity more generally, appears to be a valid strategy for marking risk of NSSI. Indicators of multiple broad forms of adversity have been shown cross-sectionally to elucidate larger effect sizes for predicting NSSI, compared to narrow measures of adversity [32]. Moreover, general measures conflate a range of adverse experiences that are likely associated with NSSI as opposed to specific events and so may better capture cumulative exposure [25]. However, broad measures of adversity such as the 15 single-item variables utilised by [32] focus uniquely on adverse event exposure, overlooking the importance of structural exposures to adversity such as availability of financial resources.

One such general assay of environmental adversity, measuring both adverse event exposure and structural adversity is the ‘environmental suppressors’ (ES) subscale of the Reward Probability Index (RPI) [35], which was shown to have internal consistency in a recent confirmatory factor analysis (CFA) [34]. The ES subscale has been evidenced as showing a moderate, unique positive correlation with ACE scores suggesting convergent validity [36]. Additionally, high ES scores are associated with lower ratings of subjective socioeconomic status (SES), a measure of monetary and opportunity wealth, indicating convergent validity with other assays of adverse environmental exposures [36,37]. Thus, while validation of the ES as a maker of adversity is in its infancy, the ES subscale may be a valid measure of cumulative general adverse environmental exposure. However, to the authors knowledge the ES has not been explored in relation to NSSI. The current study aims to assess if the ES subscale is associated with NSSI engagement, as a general recent environmental adversity index.

An additional consideration in exploring correlates and predictors of NSSI is that mediating variables remain unclear. A recent review of 25 studies assessing candidate mediators noted significant heterogeneity across studies regarding mediators identified including psychiatric (e.g., depression, anxiety, borderline personality disorder [BPD], substance misuse), psychological (e.g., self-esteem), and social factors (e.g., interpersonal difficulties) [38]. Despite variation in proposed mediators, recent reviews have established mood disorders, including depression and anxiety, and emotional dysregulation disorders characterised by impulsivity such as BPD as key risk factors for young people who engage in NSSI [17,39,40]. Longitudinal studies have found depression and anxiety, alongside alcohol and cannabis use to be independently associated with incidence self-harm [41]. Moreover, an integrative review of risk factors for NSSI in adult populations established across 52 studies, depression was the most common risk factor, followed by anxiety [42].

Longitudinal studies provide support for depression and anxiety as candidate mediators. A systematic review of 32 longitudinal studies of NSSI and deliberate self-harm, demonstrated previous history of NSSI, female gender, depression, suicidality, and psychological distress as key predictors for subsequent engagement in NSSI [39]. Longitudinal daily diary assessments have shown increased negative affect is associated with a subsequently greater likelihood of NSSI engagement and urges [43]. Furthermore, anxiety and experience of acute stressors prior to hospital presentation with NSSI have been identified as longitudinal predictors of both future NSSI and an increased likelihood of recurring presentation to hospital [44]. Specifically, findings from 4,799 participants in the Avon Longitudinal Study of Parents and Children (ALSPAC) suggest individuals engaging in NSSI had 2.63 times higher likelihood of depression, and 2.06 times higher likelihood of anxiety disorders, compared to those who did not engage in NSSI [45].

In contrast, evidence for impulsivity as a unique mediator is unclear. Impulsivity, as measured by the Barratt Impulsiveness Scale (BIS) was associated with NSSI in a meta-analysis of four studies, and indicated impulsivity was significantly higher for individuals engaging in NSSI, compared to those who do not [17]. Moreover, impulsivity measured using the BIS has been shown to be a unique prospective predictor of NSSI onset in young adults [46]. However, in other studies, when additional risk factors have been included (e.g., distress) the impulsivity-NSSI association is attenuated [47,48]. Overall, the unique, relative contribution of anxiety versus depression versus impulsivity to NSSI is unclear. Although the preponderance of published effects might point to depressive symptoms being a stronger mediator of NSSI than anxiety symptoms and impulsivity in adjusted models, the question remains as to how strong any unique association would be.

Taken together, both theoretical and empirical accounts support a risk pathway from distal factors, such as environmental adversity, to development of proximal risks, such as affective disorders and potentially impulsivity, and subsequently NSSI engagement. The main aim of the current study is to validate the environmental adversity (ES) subscale of the Reward Probability Index (RPI) as a correlate of NSSI (and related risk factors: depression, anxiety and impulsivity) to demonstrate the utility of this short ES questionnaire in self-harm research. To do this, the present study aims to test a particular model, in which environmental adversity, indexed by the ES subscale of the RPI is associated with depression, anxiety, or impulsivity, which in turn are associated with NSSI. Moreover, the current study aims to establish if environmental adversity correlates with subjective SES to provide validation of the ES subscale of the RPI as an index of cumulative social and structural adversity. There is a paucity of studies assessing these risk pathways in a single model making inference around candidate unique risk pathways difficult [49–52]. Consequently, we conducted an exploratory robust parallel path model to test whether the relationship between environmental adversity and NSSI would be mediated by depression, anxiety, or impulsivity, to assess the unique contribution of each predictor. It is predicted, in accordance with literature, depression will uniquely mediate the relationship between environmental adversity and NSSI, but it is unknown whether anxiety and impulsivity will have any unique residual mediating roles.

## Method

### Participants and design

A total of 379 participants were recruited via non-purposive sampling through advertisements on the Psychology research pool at Exeter and via social media (e.g., “Overheard at Exeter Facebook page”), and fully completed all key variables in an online, correlational survey via Qualtrics.com. The study was advertised as a project assessing ‘Mental Imagery and Distractibility’, with no mention of self-harm in any of the recruitment materials, participant information sheet or consent form. Participants were told there would be a task followed by a series of questionnaires which may ask for sensitive information pertaining to their mental health. The only inclusion criteria were participants had to be aged 18–25, and currently at university. Participants were reimbursed with course credits or a £3 Amazon voucher depending on their preference. Recruitment of the sample took place from 20^th^ November 2020–11^th^ February 2021. Participants were excluded if they did not complete 100% of the survey items (*N* = 203) or scored <80% accuracy on attention check questions which could indicate poor engagement with the survey reducing data quality (*N* = 10) [53], or not falling within the age boundaries of 18–25 applied to capture ‘young adults’ (*N* = 15). There were five attention checks distributed throughout the survey, all of which were instructed response items, to increase the likelihood of identifying inattentive or inauthentic responses [54] e.g., “Please select strongly agree for this item”. Gender was limited to male or female, due to an inadequate number of ‘prefer to self-identify’ responses (*N* = 2) for analysis, resulting in an analytic sample of 149 (83%) participants.

Of the analytic sample, 28 (18.8%) were males and 121 (81.2%) were females, age ranged from 18–25 (*M* = 20.42; *SD* = 1.39). Individuals who endorsed at least one-item on the direct self-injury subscale were considered to have engaged in NSSI over the past year (n = 75, 50.3% of the sample), those who endorsed zero items on the direct self-injury subscale were considered as ‘no NSSI controls’, hereafter referred to as controls (n = 74, 49.7% of the sample).

### Questionnaires

Three single-item questions assessed demographics. Age was assessed using an open text response, gender was assessed using a categorical question with three levels (male, female, prefer to self-identify). The MacArthur Scale of Subjective Social Status [55] was used to assess subjective socioeconomic status (SES). A single item stating “Imagine the scale below represents how society is set up. On the left are the people who are best off in terms of money, schooling, jobs, and respect. On the right are the people who are worst off. Please tell us where you think the family you grew up in falls on this scale” was shown above a visual of a ladder. Responses are ranked on a scale of 1 “worst off family” to 9 “best off family”, with lower scores notionally capturing experience of relative family poverty.

**Fig 1:**
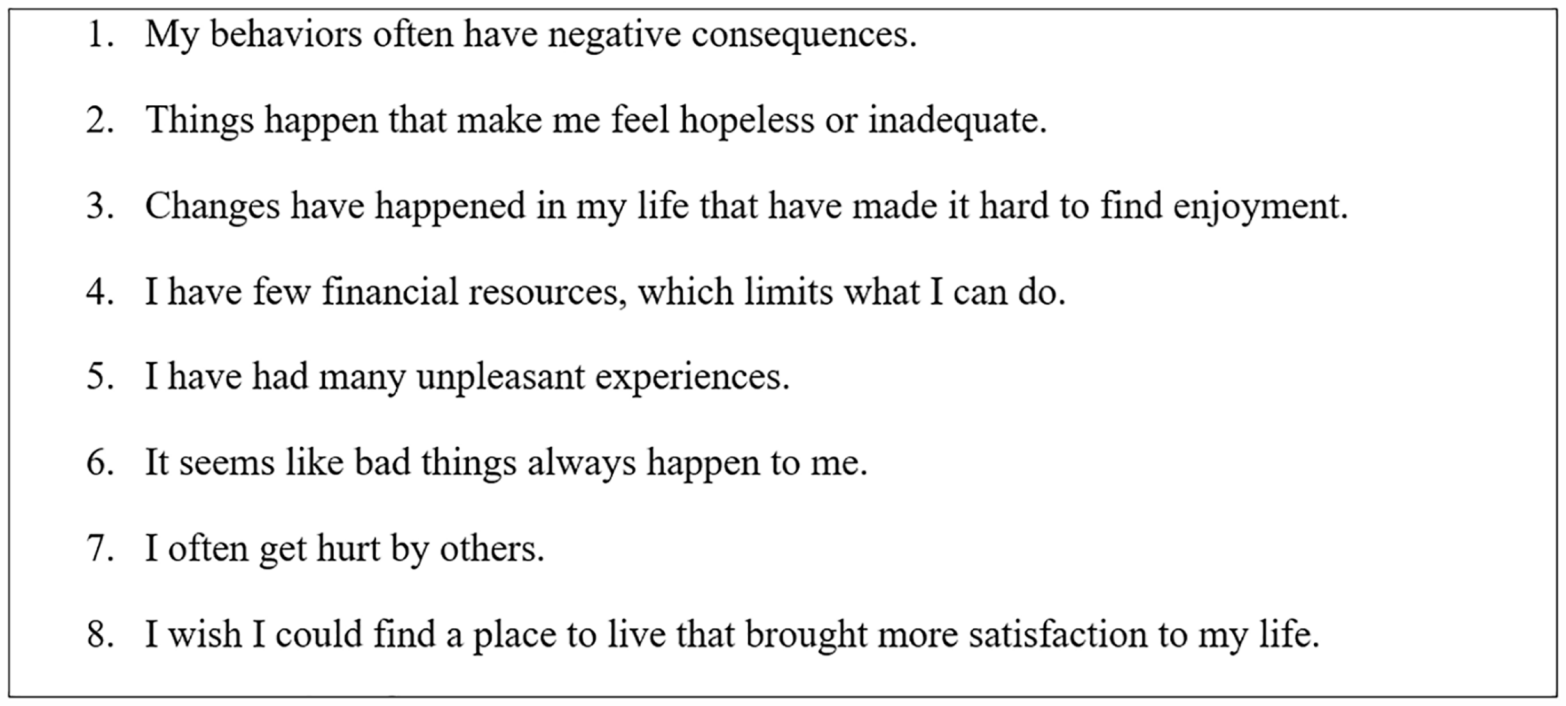
The eight items comprising the environmental suppressors subscale of the RPI, taken from the revised factor structure [34]. The total score across items represents ‘environmental adversity’ in the present study.

The Reward Probability Index (RPI) [35] environmental suppressors subscale was used to measure environmental adversity. Eight items from the revised factor structure of the RPI [34] assessed negative events and perceived environmental reward deprivation (e.g., “It seems like bad things always happen to me”). Responses were scored from 1 (“Strongly Disagree”) to 4 (“Strongly Agree”). Total scores can range from 1–4. In the current study, Cronbach’s α = .811 indicating good internal consistency, similarly to prior estimates ~ .84 [34,56]. See Fig 1 for all items of the subscale.

Depression symptoms were assessed using the Patient Health Questionnaire Depression Scale (PHQ-8 depression) [57]. Eight items relating to feelings of depression in the past 2 weeks (e.g., “Poor appetite or overeating”) were rated on a 4-point scale from 0 (“Not at all”) to 3 (“Nearly every day”). Total scores can range from 0–24. A score of 10 is the cut-off point for current depression. In the current study, Cronbach’s α = .849 indicating good internal consistency [56].

Anxiety symptoms were assessed using the Generalised Anxiety Disorder Questionnaire (GAD-7 anxiety) [58]. Seven items relating to feelings of anxiety in the past two weeks (e.g., “feeling nervous, anxious or on edge”) are rated on a 4-point scale from 0 (“Not at all”) to 3 (“Nearly every day”). Total scores can range from 0–21. Scores of 5, 10 and 15 are cut-off points for mild, moderate and severe anxiety, respectively. In the current study, Cronbach’s α = .897 indicating good internal consistency [56].

Impulsivity was assessed using the Barratt Impulsiveness Scale short form (BIS-15) [59]. 15 items assess impulsivity across three subscales; motor impulsivity (e.g., “I act on impulse”), non-planning (e.g., “I save regularly”) and attention impulsivity (e.g., “I don’t pay attention”). Responses are rated on a 4-point scale from 1 (“Rarely/Never”) to 4 (“Almost Always”). The current study utilised a total score summed across all three subscales to reflect overall impulsivity. The BIS total score has demonstrated criterion validity in discriminating between high and low frequency impulsive behaviours in samples reporting alcohol use, impulsive eating, and psychopathology [60]. In the current study, Cronbach’s α = .729 indicating acceptable internal consistency [56].

Alcohol use was assessed using the Alcohol Use Disorder Identification Test (AUDIT) [61]. 10 items assess alcohol consumption (e.g., “How often do you have a drink containing alcohol?”) and alcohol problems (e.g., “How often during the last year have you found that you were not able to stop drinking once you had started?”) over the past 12 months. Total scores range from 0–40, which was used in the analyses to indicate alcohol dependence severity (hereafter ‘alcohol use’).

Cannabis use was assessed using the Cannabis Use Disorder Identification Test Revised (CUDIT-R) [62]. Eight items assess cannabis consumption (e.g., “How often do you use cannabis?”) and cannabis problems (e.g., “How often during the past 6 months did you find that you were not able to stop using cannabis once you had started?”) over the past six months. Total scores range from 0–32, which was used in the analyses to indicate cannabis dependence severity (hereafter ‘cannabis use’).

The Direct and Indirect Self-Harm Inventory (DISH) [63] was used to assess high-risk, indirect, and direct self-harm (NSSI) behaviours engaged in over the past year. To try and ensure only behaviours with non-suicidal motives were captured, participants were provided instructions as follows: “For each of the behaviours listed below, tell us if in the last year (12 months) you have intentionally (on purpose) injured yourself without wanting to die by checking the box next to the statement. Please do not include anything that was an accident or was done with the goal of killing yourself. If you did not do the behaviour in the last year, select no.” Five items assessed high-risk behaviours (e.g., “driving recklessly and at high speeds”), four items assessed indirect NSSI behaviour (e.g., “punching walls”), and eight items assessed direct NSSI behaviours (e.g., “cut/carve skin”). The behaviour categories are reported by answering “Yes” or “No” to “Have you engaged in any of the following in the past year?”. Responses were summed across items in each subscale to produce a continuous score reflecting the number of NSSI behaviours engaged in. The continuous score yielded from the direct NSSI subscale is the outcome in the NSSI subsample analyses in the current study. The wording of the DISH was deliberately written to avoid stigmatizing language to promote accurate reporting [64,65]. Finally, the DISH has shown good convergent validity with clinical measures of self-harm behaviours such as the Self-Injurious Thoughts and Behaviours Interview (SITBI) [63,66].

### Procedures

This study was approved by the University of Exeter Psychology Ethics Committee (eCLESPsy001789 3.3). Upon accessing the link to the survey, participants were provided an information sheet and consent form to indicate their informed consent. Written consent was indicated by selecting yes to a series of questions, and selecting yes to ‘Do you consent to take part in this survey?’. If no was selected to consent, the survey automatically terminated. The survey was anonymised by removing the IP address tracking, and collecting no personally identifiable information (e.g., names or email addresses). After the survey participants were provided with a written debrief document outlining sources of support for a range of mental health difficulties (e.g., depression, anxiety) and NSSI including community (e.g., Samaritans) and app-based (e.g., StayAlive) resources.

### Data analysis

IBM SPSS v.28 was used for assumption checks, logistic regression, bivariate correlations, and descriptive statistics. Logistic regressions were conducted to assess which variables were associated with reporting past year NSSI, with gender entered as a categorical variable. The outcome variable was the binarized variable for NSSI where reporting NSSI was categorized as 1 and not reporting NSSI was categorized as 0. In the NSSI subsample (*N* = 75), assumptions for multiple regression models were checked and met with respect to no multicollinearity indicated by VIF scores <10 [67], independence of residuals indicated by Durbin-Watson values ~2 [68], and no influential cases biasing the models indicated by Cook’s distance <1 [69]. Homoscedasticity was tested using a Spearman correlation between standardized predicted values and standardized absolute residuals and was met for the path model assessing the parallel mediation paths of anxiety and depression between aversive experience and NSSI engagement (*p* = .354). However, the continuous outcome variable of NSSI was not normally distributed, displaying a clear positive skew. RStudio v4.5.1. was used to conduct robust parallel mediation using the ‘robmed’ package [70], using MM-regression and 10,000 fast-and-robust bootstrapped confidence intervals to reduce type 1 error and improve power [71]. A standardized and unstandardized model was conducted, as robmed does not concurrently produce both coefficients. The standardized model is reported in the results. Using Green, (1991)’s rule of N > 50 + 8*m* (where *m* is the number of predictors), the current study achieves minimum power (.80) for a medium effect size (*R*^*2*^ < .13) [50+(8*3)=74] [72]; however, 75 remains a small sample size for cross-sectional robust mediation. The path model assessed whether anxiety or depression symptoms uniquely mediated the relationship between aversive experience and NSSI engagement, to characterise the incremental validity of each variable in predicting NSSI, rather than temporality of variables. Impulsivity was omitted from the path model because it showed no bivariate correlation with NSSI, and no significant group difference. The model was assessed including covariates of age, gender, indirect NSSI, and SES, the following results are inclusive of covariates. To assess residual covariance between anxiety and depression as parallel mediators in the model, the “lavaan” package was used, as robmed is unable to estimate residual covariance. Mediation ratios were calculated for each of the two indirect paths to provide a percentage of variance (*R*^*2*^) each indirect path explained between aversive experience and NSSI engagement. The ratio was calculated by dividing the unstandardized indirect path coefficient for depression and anxiety by the unstandardized total effect of the model. The ratio allows for the variance accounted for by each indirect path to be directly compared.

## Results

### Logistic regression

To test which variables were associated with reporting past year NSSI, all mental health symptoms and demographic variables were entered into a series of logistic regressions to assess crude odds, and a single model to assess adjusted odds, reported in Table 1. Adjusted odds ratios were adjusted for all 12 variables. Reporting environmental adversity was associated with 3.8 times increased odds of reporting past year NSSI. Of the mental health symptoms, only depression was significantly associated with a 1.1 times increased odds of past year NSSI, with anxiety and impulsivity being non-significant. Indirect self-harm behaviours were associated with a 3.7 times increased odds of reporting past year NSSI, whereas high risk behaviours were not significant. Neither alcohol nor cannabis use were significantly associated with NSSI after adjusting for all variables. Finally, age and gender were not significantly associated with past year NSSI, however lower SES was associated with a 1.4 times increased odds of reporting past year NSSI in the adjusted model.

**Table 1:** Logistic regression showing crude and adjusted odds for all demographic and mental health variables with NSSI presence as the outcome. Adjusted odds are adjusted for all 12 variables.

	Crude Odds Ratio	*p*	Exponentiated 95% CI		Adjusted Odds Ratio	*p*	Exponentiated 95% CI	
**Environmental Adversity**	**3.931**	**<.001**	**1.985**	**7.785**	**3.835**	**.013**	**1.334**	**11.019**
**Depression**	**1.126**	**<.001**	**1.050**	**1.208**	**1.142**	**.027**	**1.015**	**1.284**
Anxiety	1.070	.041	1.003	1.142	.932	.226	.831	1.045
Impulsivity	1.044	.127	.988	1.103	.966	.370	.896	1.042
High Risk Behaviours	1.520	.002	1.168	1.978	1.186	.344	.833	1.690
**Indirect Self-Harm Behaviours**	**3.449**	**<.001**	**1.877**	**6.335**	**3.742**	**<.001**	**1.750**	**8.000**
Alcohol Use	1.032	.312	.971	1.098	1.007	.863	.929	1.092
Cannabis Use	1.245	.023	1.030	1.505	1.203	.123	.951	1.522
Age	.852	.187	.672	1.080	.826	.229	.604	1.128
Gender^a^	.852	.704	.374	1.943	1.939	.280	.583	6.456
SES	1.057	.619	.850	1.313	1.425	.015	1.052	1.931

^a^ = Reference category for gender was males (1). CI = Confidence Interval. Emboldened variables were significant in crude and adjusted models.

### NSSI subgroup analyses

The NSSI group was isolated to examine predictors of NSSI (*N* = 75). Picking and scrape/scratching skin was endorsed by over half the sample, followed by burning, preventing wounds from healing and cut/carving skin. Insertion of sharp objects into skin/nails and head banging were the least endorsed methods. Table 2 shows the prevalence of endorsed deliberate NSSI methods in the NSSI sample.

**Table 2:** NSSI in the previous year (*N* = 75).

Direct Self-Harm Inventory subscale (DISH) item number and abridged content	Prevalence (%)
1. Cut/carve skin	26.7
2. Burned Self	34.7
3. Insert Sharp Objects into skin/nails	10.7
4. Picking	57.3
5. Self-Hitting	17.3
6. Head banging	10.7
7. Scrape/scratch skin	56.0
8. Prevent wounds from healing	30.7

### Bivariate correlations

Bonferroni Correction for multiple testing was conducted (*p*-value adjusted to 0.05/12 = .004). As impulsivity did not significantly differ either between groups, nor was significantly associated with NSSI engagement it was removed from subsequent analyses. Alcohol and cannabis use were also removed for the same reason. The expected pattern of correlations was supported with the corrected significance threshold, whereby environmental adversity correlated with depression and anxiety, but only depression correlated with NSSI suggesting depression may be the key mediator. Although anxiety did not correlate with NSSI at the corrected threshold, it was retained in the path model as it may be theoretically meaningful as a component of ‘negative affect’. To allow for specificity in the model, and to account for shared variance between depression and anxiety, anxiety was retained. Descriptive statistics and bivariate correlations among the variables are summarized in Table 3.

**Table 3:** Bivariate correlations and descriptive statistics in the NSSI subsample.

	1	2	3	4	5	6	7	8	9	10	11	12
1. Environmental Adversity	--											
2. Depression	**.480** ^**^	--										
3. Anxiety	**.488** ^**^	**.680** ^**^	--									
4. Impulsivity	0.046	0.058	−0.075	--								
5. Direct NSSI	.264^*^	**.404** ^**^	.283^*^	0.148	--							
6. Indirect NSSI	0.111	−0.022	−0.070	.263^*^	0.165	--						
7. High Risk	0.125	−0.016	−0.151	0.168	0.098	.341^**^	--					
8. Alcohol Use	−0.099	0.036	−0.122	0.210	0.110	0.009	**.418** ^**^	--				
9. Cannabis Use	0.070	−0.041	−0.124	−0.074	0.119	−0.025	**.340** ^**^	0.191	--			
10. Age	−0.153	−0.041	−0.034	−0.043	−.299^**^	−0.057	0.080	0.123	0.204	--		
11. Gender^a^	0.073	0.044	0.146	−0.122	0.126	**−.395** ^**^	−.266^*^	−0.155	−.232^*^	−.227^*^	--	
12. SES	**−.419** ^**^	−0.218	−0.206	−0.012	−0.018	−0.168	0.059	0.172	−0.090	0.122	0.141	--
Mean	2.35	8.79	8.04	36.19	2.44	0.72	1.61	9.04	5.50	20.27	1.80	6.22
SD	0.48	5.14	4.96	5.58	1.49	0.78	1.36	1.36	3.13	1.33	0.40	1.50
Range	1.25-3.25	0-24	0-21	20-48	1-7	0-3	0-5	0-24	0-15	18-25	F/M(60/15)	2-9

*Note.* a = Gender ratio was reported to two categories for gender, with males as the reference category due to insufficient data (*N* = 2) in the category of “prefer to self-identify”.

All unadjusted values significance indicated by *=*p*<.05, **p=<.01 ***p=<.001.

Values emboldened remained significant following Bonferroni corrections (*p* < .004).

*N* = 75 for all variables, other than SES where *N* = 74 due to one missing value.

### Robust mediation

Following corrections for multiple tests, only depression remained significantly associated with NSSI. However, unique associations between aversive experience, anxiety, depression, and NSSI were tested in a single model as the variables are controlled simultaneously to allow unique pathways to be established [73]. Robust mediation analysis allows for the amount of variance explained by each path to be characterised, allowing statistical inference on the incremental validity of predictors and mediators. Additionally, by using robust estimation methods, non-normality in the outcome variable of NSSI does not influence the model stability. The path model used robust bootstrapping to reduce Type 1 error resulting from multiple comparisons [71].

**Fig 2:**
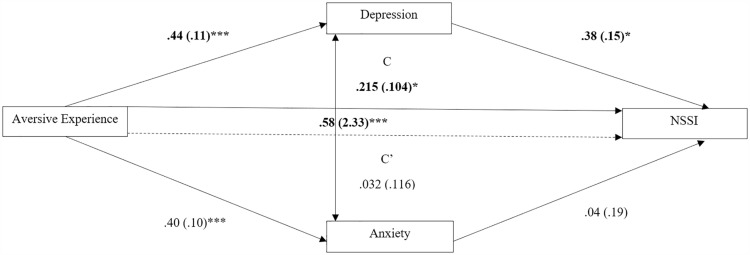
Robust mediation path model for NSSI only subsample (*N* = 75). For each connecting line, the bootstrapped standardized beta value between the two variables is shown. The 10,000-percentile bootstrapped standard error of each beta value is shown in brackets. Significant beta values are labelled as *p < .05, **p < .01, ***p < .001. Significant indirect path values are displayed as emboldened. Only depression significantly fully mediated the relationship between aversive experience and NSSI engagement, explaining 76.87% of total effects, anxiety did not mediate the relationship and explained 8.07% of total effects. Residual covariance between depression and anxiety is displayed on the bidirectional arrow between the mediators.

Fig 2 shows the standardized path model. There was a significant total effect (C path) of environmental adversity on NSSI engagement. However, when accounting for depression symptoms as a mediator, the indirect pathway (C’ path) was not significant. Depression symptoms uniquely mediated the relationship, illustrated by a significant indirect pathway via depression β = .165, 95%CI [.033,.336]. Whereas anxiety did not display a significant indirect pathway β = .017, 95%CI [−.143,.173]. This indicates depression, but not anxiety, mediated the relationship between environmental adversity and NSSI. Mediation ratio’s (*R*^2^ values) show depression explained 76.87%, whereas anxiety only explained 8.07% of the relationship between environmental adversity and NSSI.

## Discussion

The present study aimed to assess if a general index of environmental adversity predicted NSSI engagement, and whether three commonly reported risk factors; depression, anxiety, and impulsivity, uniquely mediated this relationship. The novel finding in the present study was that our general index of environmental adversity (the ES subscale of the RPI) was associated with NSSI and SES, corroborating other studies reporting associations between specific aversive experiences such as financial distress, physical, emotional, and sexual abuse, and NSSI engagement [27–29]. Moreover, the general index of environmental adversity may be advantageous, given that it does not assume a specific event exposure, and accounts for structural sources of adversity, reflecting the heterogeneity in aversive exposures individuals reporting NSSI experience [25]. The association between the ES subscale and subjective SES indicates preliminary validity for the ES subscale as a general index of external adversity. The second key finding was that depression mediated a substantial proportion of the relationship between environmental adversity and NSSI in a sample of young adults. In addition, impulsivity, alcohol, or cannabis use did not correlate with environmental adversity, anxiety, depression or NSSI indicating they were not associated with any risk factors of NSSI in the current sample. Utilising robust parallel mediation to explore the contribution of depression and anxiety revealed depression symptoms uniquely mediated the greatest proportion of variance of NSSI in individuals exposed to aversive experiences, distinct from anxious symptoms. These findings corroborate published systematic reviews which suggest that depression is the most common cross-sectional risk factor [42], and the strongest prospective risk factor for NSSI [39,45]. Furthermore, the findings corroborate evidence that impulsivity does not uniquely predict NSSI when additional risk factors, such as depression are included in the model [47,48]. The key contribution of this finding is that assessing all risk factors together, in a single cross-sectional model allows delineation of which factor explains the most variance, thus providing evidence for the validity of specific risk factors. The main insight from the present study is that developing and validating metrics of environmental adversity is key in advancing models of NSSI risk. Moreover, depression symptoms appear, at least cross-sectionally, to be important unique markers of risk for NSSI engagement, over symptoms of anxiety or impulsivity. Thus, interventions for NSSI engagement should consider both environmental exposures to adversity, and depressive symptoms as targets.

The current findings support the affect regulation accounts of NSSI (e.g., EAM) [11], by demonstrating negative affect (i.e., depression) as the unique mediator of the adversity-NSSI pathway. The current study extends on these models by identifying depressive symptoms as uniquely associated with NSSI engagement, above other negative affect symptoms such as anxiety. Moreover, the findings support more complex sequential models (e.g., Integrated Model of NSSI) [13] utilising a ‘diathesis-stress’ approach to explaining NSSI engagement. In this type of model, adverse experience would be a distal predictor of NSSI engagement, which is mediated by depressive symptoms, providing aversive experience exposure as the ‘diathesis’ of NSSI, and depressive symptoms as a potential stress response, subsequently leading to NSSI engagement. Though this type of model requires testing using longitudinal data.

Given the cross-sectional nature of the present study, it must be considered that this pathway may operate in reverse. In that, NSSI engagement may influence depressive symptoms and environmental adversity as a predictor rather than an outcome. It is plausible that engaging in NSSI may make an individual feel worse, and as a result they may perceive their environment as more adverse. The use of a cross-sectional design precludes the ability to draw robust conclusions regarding the temporality of the risk sequence. Previous findings have indicated that the evidence for NSSI as a prospective predictor of depression are mixed, though preliminary meta-analytic investigations suggest NSSI engagement does not prospectively predict depression [74]. Further research utilising short (e.g., days and weeks) and long-term (e.g., months and years) follow-up periods is required to disentangle the relationship between NSSI and depressive symptoms across time. Thus, the temporality of the risk sequence remains unclear and is an area requiring further study.

Utilising a broad measure of environmental adversity via the ES subscale in the current study captured the overall experience of adversity, rather than specific events, suggesting any adverse experience exposure may be important in explaining NSSI engagement. This reflects growing knowledge that broad and heterogeneous exposures to adverse events and environments are important in predicting risk of NSSI engagement, and may be related to risk of developing depression, thereby influencing depression-related behaviours [25,75]. While the ES appears to be a valid marker of risk for NSSI in the present study, assessment of its convergent validity with other markers of adversity, such as the LE-C is needed [27]. It is unclear whether the items on the ES are directly assessing adverse experience as a consequence of transaction with the environment, or if they may be assessing access to different environmental exposures, or perceptions of adversity. Thus, further corroboration with existing measures of adversity is needed to establish the construct validity of ES as a broad index of environmental adversity, particularly in samples reporting NSSI. Another avenue for future research could be to conduct daily diary studies to assess the diversity and accumulation of adverse exposures over time. Notwithstanding the further validation required, the predictive validity of the ES subscale in the present study implies that in looking at risk factors for NSSI engagement, and in turn, ways to reduce NSSI engagement, external factors should be considered. Considering external influences as a target for intervention diverges from the common approach in the literature which is to suggest a reduction of individual-level symptoms. This may involve assessing the environment of individuals engaging in NSSI to find solutions to reduce adversity as far as possible, rather than targeting symptom reduction alone. In clinical settings, the ES subscale may be useful as a brief general assay of perceptions of adversity as a broader, less intrusive measure than traditional ACE inventories, and/or a more complete assay alongside them. Higher scores on the ES may indicate a cognitive disposition towards perceived adversity, which may be a barrier, or area of interest during treatment. Furthermore, the ES provides a brief assessment of current perceptions of adverse exposures, as opposed to a retrospective report of prior adversity, which may be more relevant in guiding treatment decisions and support offered [76].

The current study did not corroborate previous research showing anxiety [42,44] and impulsivity [17,46] are uniquely associated with NSSI engagement. The lack of association observed for anxiety may be explained by the high covariance between anxiety and depression symptoms, whereby anxiety does not contribute unique explanation of variance for NSSI, when depression is included simultaneously. Previous studies [41] utilising aggregate scores of depression and anxiety would not illustrate this distinction. Thus, one implication of the present study is to avoid aggregating depression and anxiety measures as there may be unique paths to NSSI engagement. However, it should be noted that the current study did not assess clinical depression or anxiety, simply symptoms of both assessed using standardized measures. Therefore, it is possible that the present findings may not generalise to samples expressing symptoms meeting or exceeding clinical thresholds on either scale.

In terms of impulsivity, the present study used the BIS total score, which measures impulsivity across three facets; Cognitive, Motor, and Non-Planning [59]. Importantly, Barratt (1993) [77] developed the BIS with the assumption that impulsivity is independent (or ‘orthogonal’) of emotional states, therefore, the BIS total score only reflects difficulty planning for the future, dysregulation of physical impulses, and executive dysfunction such as difficulty making decisions. In other words, the BIS conceptualises impulsivity as a trait of ‘non-planning’. While the BIS has been associated with NSSI both cross-sectionally [17] and as a prospective predictor [46], other conceptualisations of impulsivity acknowledge the role of emotion in the facet of ‘urgency’ which refers to an immediate need to resolve a strong emotional state. The Theory of Urgency proposes maladaptive behaviours (e.g., NSSI, substance use) result from increased urgency (i.e., impulsiveness) to immediately alleviate highly emotional states, despite threats to long-term goals such as health [78]. Measures such as the “Negative Urgency, (lack of) Premeditation, (lack of) Perseverance, Sensation Seeking, Positive Urgency,” or “UPPS/UPPS-P” include facets of urgency, alongside more ‘cognitive’ aspects such as premeditation and perseverance to capture all relevant facets of impulsivity across different theoretical approaches [79]. Previous research indicates the importance of different facets of impulsivity in cross-sectional studies assessing multiple risk factors for NSSI, in that ‘negative urgency’ is the most predictive facet of impulsivity in assessments of cross-sectional risk [80]. Additionally, based on a meta-analysis assessing the UPPS in over 40,000 participants negative urgency was the strongest facet associated with a broad range of psychopathology, including NSSI [81]. However, it should be noted that for NSSI, negative urgency was not significantly more predictive than the other facets measured by the UPPS, suggesting multiple facets of impulsivity may be relevant in predicting NSSI. Given that the BIS does not assess for negative urgency, future research should utilise measures such as the UPPS to assess ‘emotional’ facets of impulsivity in relation to NSSI.

Another explanation for the failure to detect a unique contribution of anxiety or impulsivity may be that the current study utilised a monolithic conceptualisation of NSSI by assessing the total scores of the DISH ‘direct’ subscale. While the DISH provides benefits in brevity and lack of judgemental language to encourage reporting of NSSI [63–65], compared to other commonly-used measures of NSSI such as the Self-Injurious Thoughts and Behaviour Interview (SITBI) [66] and the Alexian Brothers Assessment of Self-Injury (ABASI) [82], the subscale includes a narrow range of behaviours constituting NSSI. Moreover, the DISH provides no index of frequency to establish the ‘severity’ of NSSI engagement, nor ‘motives’ for engaging in NSSI. As impulsivity [17,18] and anxiety symptoms [25,83] are frequently reported as having strong associations with repeated NSSI engagement, the lack of a frequency index of NSSI in the current study may explain why no relationship was observed. It is unclear whether, in the current sample, individuals were reporting one acute NSSI event in the past year, or recurring NSSI behaviours. This is further limited by a lack of information on the reliability of the DISH as a measure of NSSI, noted by the scale authors as an area for future study [63]. The present study was unable to assess test-retest reliability of the DISH due to the cross-sectional design, and item-level analyses were precluded by the small sample size offering inadequate statistical power. Thus, future studies should include an index of frequency to establish if associations between impulsivity, anxiety symptoms, and NSSI are moderated by NSSI engagement frequency, and if frequency affects the observed mediational path via depression symptoms. Moreover, the test-retest reliability of the DISH should be established by assessing the measure in longitudinal studies.

Another consideration is that despite not referencing self-harm in any of the study advertising, the prevalence of NSSI in the current study being 50% is very high. There are several potential explanations for the high prevalence. Firstly, as participants were recruited using the University Psychology research participation pool, the students were likely majority psychology students. Psychology students may be more mental health literate compared to other students, and as such, may be more likely to report symptoms [84]. Indeed, across prior studies assessing NSSI in non-representative samples of psychology students, prevalence varies widely from 5–55% [85]. Additionally, the sample were predominantly female. There is evidence that female students, particularly those in disciplines such as medicine and psychology, may hold fewer stigmatizing attitudes towards mental disorders, and as such may be more willing to disclose their own symptoms [86]. Alternatively, the high prevalence may be due to the low threshold used for classifying past year NSSI engagement. Participants were required to score one or above on the DISH to be considered in the NSSI group, this included those who only endorsed items such as picking or scratching. Endorsement of these items alone has previously prevented individuals from being classified as engaging in NSSI, as it is assumed that in the milder forms, picking skin and scratching would not cause tissue damage. However, these behaviours do commonly occur in more severe forms, and are recognised as such in popular NSSI measures such as the ABASI in items describing ‘picking at wounds’ [82]. Given that the intent to cause oneself harm is the key criterion for classifying self-harm, and evidence that skin picking and scratching are valid forms of self-harm, we chose to retain this criteria [87]. However, we acknowledge this may have led to a much higher prevalence estimate than studies which require multiple forms of injury to classify NSSI. It is important to note the current study does not intend to provide an estimate of the population level of clinically significant self-harm, simply a description of the self-harm prevalence in a community sample of university students.

Several limitations of the current study should be noted. First, the lack of a ‘motives’ measure precludes the ability to state why participants in the current study engaged in NSSI. The motive for NSSI engagement is likely multidetermined and changeable, which may constitute an additional proximal risk modifiable via intervention [49]. Thus, while it can be concluded that depressive symptoms explained more variance than anxiety in NSSI engagement in the current study, it cannot be stated that NSSI was engaged in to ‘relieve’ depressive symptoms. The ‘function’ of NSSI engagement requires further exploration using motive subscales (e.g., criterion B on the ABASI) to explain why depressive symptoms are associated with NSSI, and if different motives for NSSI engagement affect the risk pathway. Secondly, the cross-sectional nature of the current study does not allow temporal inference of variables in the risk pathway. Our study utilises previous evidence to suggest the likely order of factors (i.e., aversive experience leading to depressive symptoms, leading to NSSI). This is further compounded by a lack of temporal sensitivity in the measures used, where the GAD and PHQ assess symptoms in the past two weeks, and NSSI may be from any time in the previous 12-months. While it is common to assess mental health symptoms, and current self-harm in this way, it means there is little granularity on identifying the temporal sequence of the variables proposed to confer risk. The current study can only establish the validity of psychometric constructs which may reflect longitudinal effects, rather than provide evidence for longitudinal effects themselves; for which prospective designs are required [88]. Thirdly, despite attempts to enhance data quality and identify inauthentic responses by using stringent inclusion criteria of 100% survey completion, and scoring above 80% on attention checks, the unsupervised online survey method for collecting data used in the present study is known to produce inattentive responses resulting in low data quality and levels of engagement [89,90]. Supervised online survey methods, or supervised epidemiological studies are required to assess if results differ depending on response method. Finally, the sample was predominantly composed of female participants, and age was restricted to 18–25 to reflect young adults, who are a high-risk group for NSSI [91]. This means our findings cannot be generalised reliably to males, and may not extend to child and adolescent samples. Gender differences in NSSI, in that females tend to report a higher prevalence while males tend to report methods with greater severity, or potential for lethality are well documented in the literature [92,93]. However, gender differences in NSSI prevalence appear to reduce with age, being widest in mid-adolescence and attenuating by early adulthood [94]. Nonetheless, using sampling methods such as quota sampling to ensure a better representation of males in future studies would be beneficial. NSSI is prevalent among children and adolescents, with similar risk variables identified such as ACE’s to capture aversive experience, mental health symptoms and substance misuse [51,95]. However, neurodevelopmental differences may increase the relative importance of different risk variables. For example, in adolescence, impulsivity may play a unique and stronger role in NSSI engagement given this developmental period is often characterized by increased impulsivity and poorer decision making than adulthood [96,97]. These differences must be carefully considered when constructing models of risk, and the current study findings should not be applied to different age ranges.

In conclusion we found the ES was a valid predictor capturing structural and social aspects of adversity, in relation to NSSI. The main contribution of the current study was demonstrating, for the first time that a general cumulative social adversity index (the ES subscale of the RPI) is associated with, and predicts NSSI engagement in a sample of young adults. Depression was the unique mediator of the relationship between aversive experience and NSSI engagement. An additional contribution of the current study was establishing depression mediates the relationship over and above anxiety and impulsivity; clarifying the role of ‘negative affect’ and ‘emotional dysregulation’ in NSSI may be centred in depressive symptoms. The findings suggest adverse environmental experiences are a distal predictor of NSSI, which supports ‘experiential’ accounts of NSSI aetiology, suggesting the root of NSSI may be in experience, rather than the individual themselves. Thus, assessments for NSSI should include an assessment of external aversive experience, as well as assessing psychiatric symptoms and NSSI behaviour. The ES subscale represents a brief, general index to assess aversive experience as a key correlate of NSSI.
